# Kalkitoxin Reduces Osteoclast Formation and Resorption and Protects against Inflammatory Bone Loss

**DOI:** 10.3390/ijms22052303

**Published:** 2021-02-25

**Authors:** Liang Li, Ming Yang, Saroj Kumar Shrestha, Hyoungsu Kim, William H. Gerwick, Yunjo Soh

**Affiliations:** 1 Department of Dental Pharmacology, School of Dentistry, Jeonbuk National University, Jeonju 54896, Korea; liliang198761@126.com (L.L.); thesaroj8@gmail.com (S.K.S.); 2 Department of Periodontology, School of Dentistry, Jeonbuk National University, Jeon-Ju 561-756, Korea; 18643573922@163.com; 3 College of Pharmacy, Ajou University, San 5, Woncheon-dong, Youngtong-gu Suwon 443-749, Korea; hkimajou@ajou.ac.kr; 4 Center for Marine Biotechnology and Biomedicine, Scripps Institution of Oceanography and Skaggs School of Pharmacy and Pharmaceutical Sciences, University of California at San Diego, La Jolla, CA 92037, USA; wgerwick@ucsd.edu; 5 Laboratory of Pharmacology, School of Pharmacy, Jeonbuk National University, Jeon-Ju 561-756, Korea

**Keywords:** kalkitoxin, marine natural product, osteoclast, inflammation, bone loss

## Abstract

Osteoclasts, bone-specified multinucleated cells produced by monocyte/macrophage, are involved in numerous bone destructive diseases such as arthritis, osteoporosis, and inflammation-induced bone loss. The osteoclast differentiation mechanism suggests a possible strategy to treat bone diseases. In this regard, we recently examined the in vivo impact of kalkitoxin (KT), a marine product obtained from the marine cyanobacterium *Moorena producens* (previously *Lyngbya majuscula*), on the macrophage colony-stimulating factor (M-CSF) and on the receptor activator of nuclear factor κB ligand (RANKL)-stimulated in vitro osteoclastogenesis and inflammation-mediated bone loss. We have now examined the molecular mechanism of KT in greater detail. KT decreased RANKL-induced bone marrow-derived macrophages (BMMs) tartrate-resistant acid phosphatase (TRAP)-multinucleated cells at a late stage. Likewise, KT suppressed RANKL-induced pit area and actin ring formation in BMM cells. Additionally, KT inhibited several RANKL-induced genes such as cathepsin K, matrix metalloproteinase (MMP-9), TRAP, and dendritic cell-specific transmembrane protein (DC-STAMP). In line with these results, RANKL stimulated both genes and protein expression of c-Fos and nuclear factor of activated T cells (NFATc1), and this was also suppressed by KT. Moreover, KT markedly decreased RANKL-induced p-ERK1/2 and p-JNK pathways at different time points. As a result, KT prevented inflammatory bone loss in mice, such as bone mineral density (BMD) and osteoclast differentiation markers. These experiments demonstrated that KT markedly inhibited osteoclast formation and inflammatory bone loss through NFATc1 and mitogen-activated protein kinase (MAPK) signaling pathways. Therefore, KT may have potential as a treatment for destructive bone diseases.

## 1. Introduction

Osteoclasts are multinucleated cells that play a crucial role in bone resorption, skeleton development, and bone regulation. The decrease in bone quality and quantity enhances the risk of bone disorders such as osteoporosis, arthritis, etc. [1,2,3]. Therefore, osteoclasts are significant targets for the development of drugs that target damaging bone diseases. Osteoclasts are formed as a result of proliferation, fusion, and differentiation of monocytes/macrophages in the presence of receptor activator nuclear factor-kB ligand (RANKL) and macrophage colony-stimulating factor (M-CSF) [4,5].

Tumor necrosis factor receptor-associated factor 6 (TRAF6) is activated by RANK and RANKL binding, resulting in the activation of intracellular responses such as NF-кB, mitogen-activated kinases (MAPK) signaling pathways, and nuclear factor of activated T cells c1 (NFATc1) pathways. In turn, these pathways are crucial for osteoclast formation [1,6,7]. NFATc1 targets genes specific to osteoclasts, such as cathepsin K, MMP9, and tartrate-resistant acid phosphatase (TRAP) [8,9], and thus NFATc1 is well known as a significant controller of osteoclastogenesis.

*Moorena producens* (previously known as *Lyngbya majuscula*) is a species of filamentous cyanobacterium found in shallow tropical marine ecosystems, and collections from Curaçao were the original source of the natural product kalkitoxin [10]. Kalkitoxin is a biologically active lipopeptide that shows tumor-selective cytotoxicity in clonogenic assays, inhibits inflammation, is ichthyotoxic and interacts with the tetrodotoxin- and voltage-sensitive sodium channel [11,12,13]. However, the molecular mechanism of kalkitoxin in osteoclast differentiation is unknown, and the impact of kalkitoxin on in vivo bone destruction is not well known. Therefore, the aim of the current study was to determine the impact and mechanism of kalkitoxin in anti-osteoclastogenesis.

## 2. Results

### 2.1. KT Reduced Osteoclastogenesis in Bone Marrow-Derived Macrophages (BMMs)

**Figure 1:**
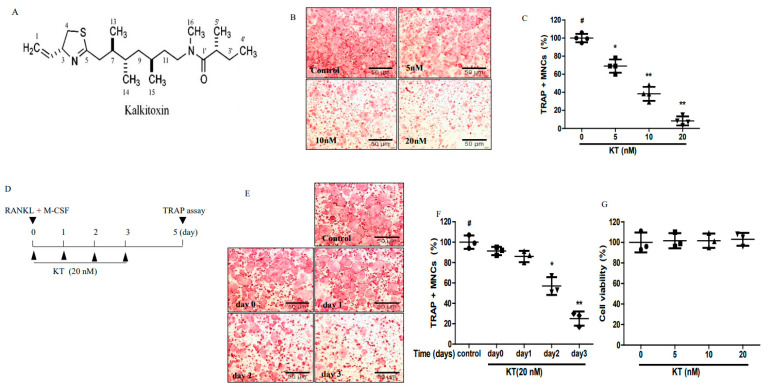
Kalkitoxin (KT) inhibits the receptor activator of nuclear factor κB ligand (RANKL)-induced osteoclast formation in bone marrow-derived macrophages (BMMs). (**A**) Chemical structure of KT. (**B**) BMMs were cultured in a 96-well plate with the macrophage colony-stimulating factor (M-CSF) (30 ng/mL) and RANKL (100 ng/mL) in the absence or presence of KT (5, 10, 20 nM) for 5 days. Tartrate-resistant acid phosphatase (TRAP) (+) multinucleated cells with three or more nuclei were counted, and (**C**) the scatter plot represents the percentage of TRAP^+^ MNC relative to the RANKL treated group. (**D**) BMM cells in the in vitro timetable. (**E**,**F**) BMMs were incubated with the indicated concentrations of M-CSF, RANKL for 5 days, and KT for 2 days, respectively and the percentage of TRAP^+^ MNC was decreased on different days relative to the RANKL treated group. (**G**) BMMs were seeded with M-CSF (30 ng/mL), treated with KT (5, 10, 20 nM) for 72 h, and the MTT assay was used to observe cell viability. Mean ± SD, *n* = 3, * *p* < 0.05, ** *p* < 0.01 vs. RANKL treated (#).

The effects of KT in osteoclastogenesis were examined. We used BMM cells to observe the effect of KTs in a dose and time-dependent manner in the in vitro osteoclastogenesis models that were treated with RANKL and M-CSF. BMMs were incubated with different concentrations of KT for 5 days with changing of the media every 2 days. Figure 1B,C showed that KT suppressed osteoclastogenesis in a concentration-dependent manner in BMMs. BMMs were treated with KT at different time points (0–3 days) and incubated for 5 days to elucidate which step of maturation of osteoclastogenensis (e.g., proliferation, differentiation, polarization, and resorption) KT is involved in. As shown in Figure 1E,F, KT markedly suppressed osteoclast cell formation from BMMs in 2–3 days. Up to day 2 and day 3 of treatment with KT, the agent affected osteoclast precursor cells and inhibited BMMs. Furthermore, the different doses of KT did not show any cytotoxic effect in BMMs (Figure 1G). Thus, KT suppressed osteoclast formation at a late stage and at non-cytotoxic doses. Interestingly, osteoblast MC-3T3E1 cells were not affected by KT.

### 2.2. KT Impairs Bone Resorption and Actin Ring Formation in BMMs

**Figure 2:**
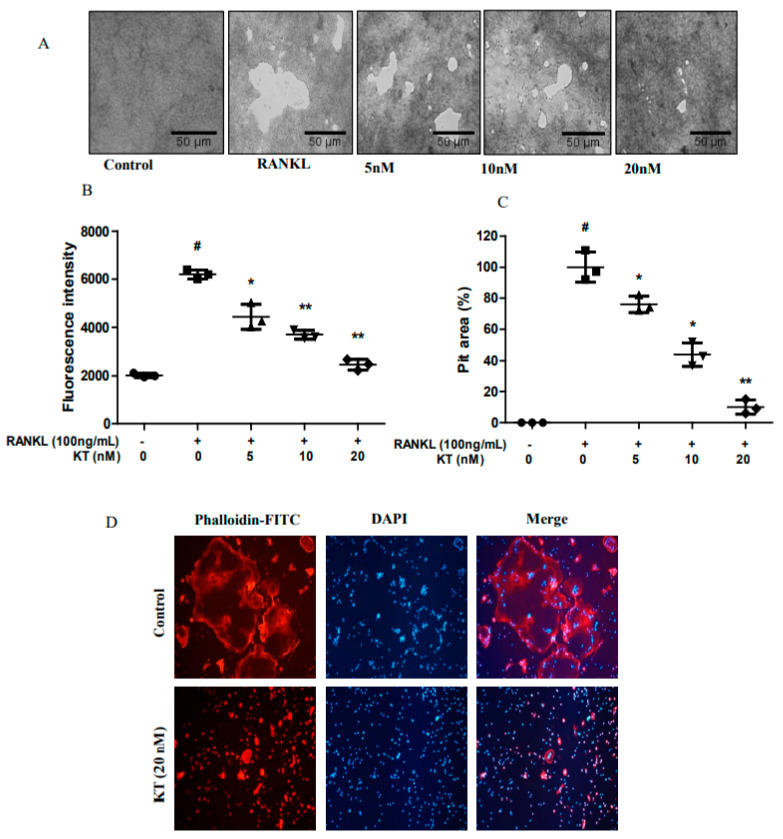
KT inhibited pit area formation and disrupted F-actin ring structure. (**A**) BMMs were differentiated in CaP-coated 48-well plates with M-CSF (30 ng/mL) and RANKL (100 ng/mL) in the absence or presence of KT (5, 10, 20 nM) for 6 days. (**B**) Fluorescence intensities of the media were observed. (**C**) Scale bar represents the percentage of pit area resulting from bone resorption. (**D**) BMM cells were seeded with M-CSF (30 ng/mL), RANKL (100 ng/mL), and 20 nM KT for 6 days, cells were fixed, and F-actin staining was performed. Mean ± SD, *n* = 3, * *p* < 0.05, ** *p* < 0.01 vs. RANKL treated (#).

Since KT repressed osteoclastogenesis, we next investigated whether KT might decrease the osteoclast bone resorption activity in calcium coated plates. Therefore, we seeded BMM cells in calcium-coated plates in the absence or presence of KT. As shown in Figure 2A, mature osteoclasts in the RANKL treated group broadly resorbed calcium phosphate in these coated plates. KT markedly decreased this resorption activity in a dose-dependent manner (Figure 2B,C). Osteoclast precursor cells were differentiated into mature osteoclasts and formed a clear actin-ring, an indication of mature osteoclasts being formed during osteoclastogenesis [14]. Next, we evaluated whether KT could suppress actin ring formation. BMMs in the RANKL treated group clearly showed the formation of an actin ring (Figure 2D, upper panel). However, the production of the actin ring was markedly inhibited by KT in BMMs. This result suggests that bone resorption and F-actin ring formation are interrelated to the formation of mature osteoclasts.

### 2.3. KT Negatively Regulates RANKL-Induced Gene and Protein Expression Levels

**Figure 3:**
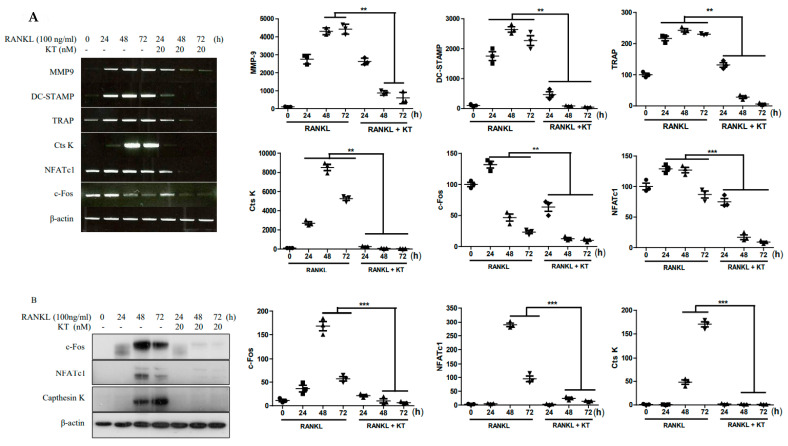
KT suppressed RANKL-stimulated gene expression and protein expression. BMMs were incubated with M-CSF (30 ng/mL) and RANKL (100 ng/mL) in the presence or absence of 20 nM KT for the number of indicated days. (**A**) Levels of MMP-9, cathepsin K, TRAP, DC-STAMP, c-Fos, and NFATc1 genes were analyzed. (**B**) The protein expression levels of cathepsin K, c-fos, and NFATc1 were performed by the immunoblot analysis. β-actin served as a reference protein. Mean ± SD, *n* = 3, ** *p* < 0.01, *** *p* < 0.001 vs. RANKL treated.

RANKL activation promotes the expression of osteoclast specific genes throughout cell differentiation [1]. Thus, BMMs were treated with KT to identify the inhibitory effects in osteoclast marker genes and protein expression. As shown in Figure 3A, the KT treatment of RANKL-stimulated BMMs significantly reduced expression of NFATc1, c-Fos, Ctsk, MMP9, DC-STAMP, and TRAP genes at various time points. Furthermore, as shown in Figure 3B, the KT treatment of RANKL-stimulated cells dramatically reduced protein levels of Ctsk, NFATc1, and c-Fos at different time points. Thus, these results suggest that KT inhibits both gene and protein expression in BMMs.

### 2.4. KT Repressed RANKL-Stimulated MAPK and AKT Pathways in BMMs

**Figure 4:**
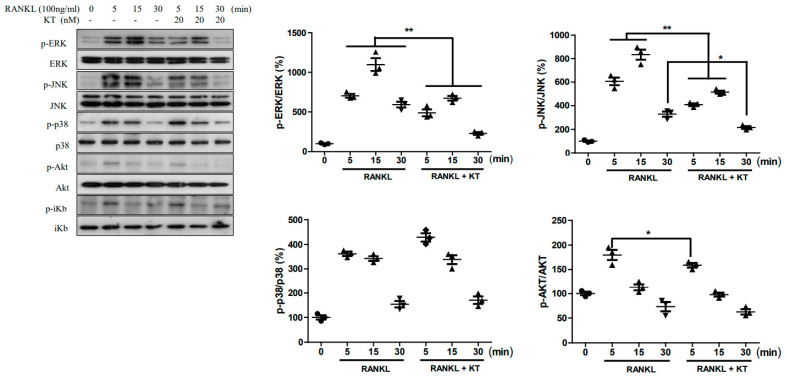
KT suppressed RANKL-stimulated MAPKs pathways. BMMs were treated for 30 min with or without KT (20 nM), after which M-CSF (30 ng/mL) and RANKL (100 ng/mL) were applied for the indicated time period. The total protein extract was used to perform an immunoblot analysis using antibodies p-p38, p-JNK, p-ERK1/2, p-AKT, p-38, JNK, ERK, and Akt. Mean ± SD, *n* = 3, * *p* < 0.05, ** *p* < 0.01 vs. RANKL treated.

RANKL stimulation of the MAPK and Akt pathways plays a crucial role in osteoclastogenesis [15,16]. Therefore, we examined the effect of KT on the MAPK pathway stimulated with RANKL in BMM cells. As shown in Figure 4, RANKL-stimulation enhanced the expression of proteins in the MAPK pathway such as ERK, JNK, and p38. The KT application to these RANKL-stimulated cells showed reduced expression of ERK and JNK, but not of p38 and AKT pathways. Thus, this result suggests that KT is able to suppress the MAPK signaling pathway and thereby prevent osteoclast differentiation.

### 2.5. KT Administration Prevents LPS-Induced Bone Loss in Mice

**Figure 5:**
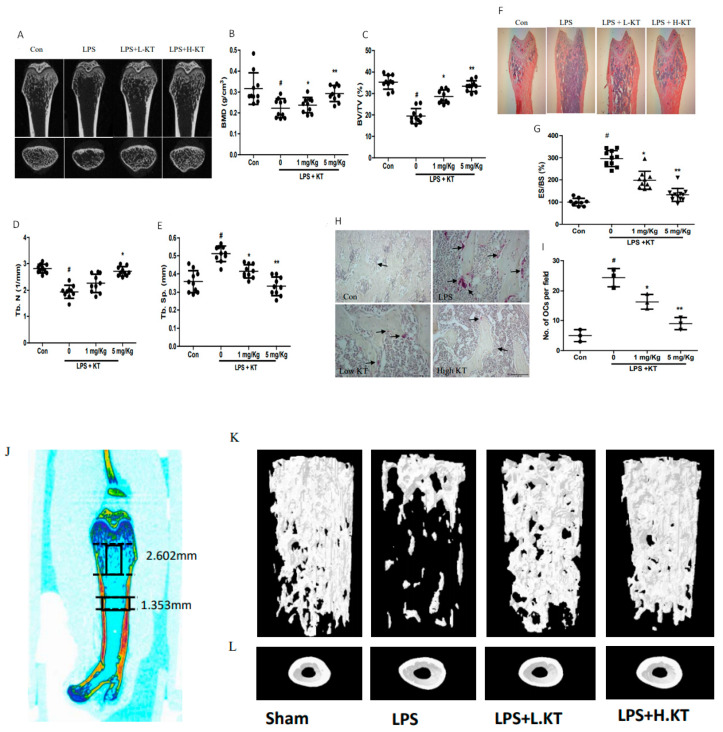
KT prevents LPS-stimulated bone loss in vivo. (**A**) LPS injected mice (6-week old) were sacrificed after 10 days, and a radiographic image of proximal femurs was achieved using a micro-CT scanner. (**B**–**E**) Bone mineral densities (BMD) and various parameters were measured by the scanner software. (**F**,**G**) The femur was fixed, decalcified, embedded, and sectioned. H&E staining was performed. The histological image showed the eroded surface of the femur. (**H**,**I**) TRAP staining was performed and osteoclast numbers in the femur were counted. (**J**) 3D µCT image of cortical and trabecular bone of the femur. Region of interest (ROI) was chosen for analysis, including cortical compartment of the femoral midshaft and trabecular compartment of distal femoral metaphyseal. (**K**,**L**) Representative 3D µCT images of vehicle treated group (Sham) and LPS, LPS with low and high KT treated groups. Mean ± SD, * *p* < 0.05, ** *p* < 0.01 vs. LPS treated (#).

As KT inhibited osteoclastogenesis in BMM cells in the in vitro experiment, we next examined the in vivo effects of KT treatment in mice. LPS was injected intraperitoneally with or without KT at specified time points (Figure 5A). From micro-CT examination, mice injected with LPS showed significantly decreased bone mineral densities (BMD), bone volume fraction, and trabecular number in femurs, which were attenuated by the KT treatment in a dose-dependent manner (Figure 5B–E). Whereas, LPS injected mice showed a notable increase in trabecular separation and eroded bone surface, this symptom was notably decreased by the KT treatment (Figure 5F–G). Cortical BMD, bone area, bone marrow area, and thickness were decreased in LPS treated mice, however, the KT treatment reversed these values. From 3D µCT image analyses (Figure 5J), the KT treatment significantly increased cortical thickness at the femoral midshaft and trabecular bone volume and thickness at the distal femoral metaphyseal (Table S1). Furthermore, the KT treatment was able to repress LPS-induced TRAP-positive cells in vivo (Figure 5H–L), suggesting that KT can prevent LPS-induced bone loss in mice.

## 3. Discussion

More than 200 million people worldwide are subject to osteoporosis with the accompanying bone loss [17]. Enhanced RANKL signaling induces progressive and excessive bone resorption, which is a hallmark of osteoporosis. Hence, suppression of downstream signals from RANKL stimulation is a feasible way to treat bone loss-related diseases [18]. Our studies first found that KT has a protective effect on bone destruction. Here, we have demonstrated that KT remarkably inhibits osteoclast formation in RANKL-induced BMMs, specifically at a late stage in vitro. KT also suppressed the induction by RANKL of osteoclastogenesis-related genes and proteins via blocking the ERK and JNK pathways.

During osteoclastic bone resorption, osteoclast precursors are recruited, adhere to the bone surface, and differentiate into mature osteoclasts. Activation of these osteoclasts induces the polarization of their cell membrane towards the bone, followed by the secretion of protons and lytic enzymes into a sealed resorption vacuole [1]. Genomic markers for osteoclastogenesis regulation such as TRAP, Cts K, and MMP9 are expressed in mature osteoclasts, known as matrix enzymes. These are responsible for the degradation of organic bone matrix [19]. As shown in Figure 3A, the inhibitory effect of KT on RANKL-induced osteoclastogenesis is likely associated with the suppression of osteoclastic specific genes, including MMP-2, MMP-9, RANK, TRAP, and Ctsk.

The transcription factors c-Fos, a member of the activator protein-1 (AP-1) family, and NFATc1 can be activated at an early stage during osteoclast differentiation. c-Fos can also be recruited to induce and activate NFATc1 [8]. RANKL stimulation leads to the activation of NF-κB and induction of NFATc1, while resulting in the expression of early responsive genes [20]. Our data showed that KT led to the decreased mRNA levels of transcription factors c-Fos and NFATc1. The protein level of c-Fos was also downregulated by KT when compared to the RANKL-induced upregulation of this transcription factor (Figure 3B). Multiple downstream signaling pathways, including MAPK, Akt, and NF-κB, are activated as the RANKL/RANK binding recruits TRAF6 [20,21]. In the present study, we demonstrated that KT inhibited the JNK and ERK phosphorylation, but not phosphorylation of p38. Therefore, it appears that KT decreases the expression of transcription factors c-Fos and NFATc1 owing to the downregulation of JNK and ERK activation by RANKL.

LPS stimulated monocyte/macrophage or macrophage precursor cells secrete several pro-inflammatory cytokines and inflammatory mediators, which in turn influences the fusion of these cells into mature osteoclasts, leading to inflammatory bone resorption [22]. In our in vivo experiments, we use an inflammatory bone resorption model through the intraperitoneal application of LPS. The results indicated that KT increases the levels of BMD, BV/TV, and Tb. N, while also decreases Tb. Sp and ES/BS compared with the LPS groups, as shown in radiographic and histological results (Figure 5 and Table S1). Our in vivo data support a previous report that showed an anti-inflammatory effect of KT, and provides initial data in the support of KT protection against osteoclastic bone resorption [23].

In summary, KT exerts profound anti-osteoclastogenic properties and can significantly inhibit RANKL-stimulated osteoclast differentiation in vitro. We also found that the suppression of signaling molecules c-Fos and NFATc1 by KT was able to block the master genes involved in regulating osteoclastogenesis expression. Furthermore, we showed that KT markedly decreased the phosphorylation of ERK and JNK in RANKL-induced osteoclasts. Finally, the in vivo data confirmed the therapeutic efficacy of KT, and suggest that KT could be developed as an alternative therapeutic drug to treat or prevent disorders associated with bone lysis.

## 4. Materials and Methods

### 4.1. Reagents

Fetal bovine serums (FBS), α-modified essential medium (α-MEM), and penicillin were purchased from Gibco (Gaithersburg, MD, USA). RANKL was purchased from PeproTech (Rocky Hill, NJ, USA), and M-CSF was purchased from R&D Systems (Minneapolis, MN, USA). All primary antibodies as phospho-p38, p38, phospho-ERK, ERK, phospho-JNK, JNK, phospho-Akt, Akt, and c-Fos were obtained from Cell signaling Technology (Danvers, MA, USA). NFATc1 and cathepsin K were obtained from Santa Cruz Biotechnology (Dallas, TX, USA). Trizol and Supercript II Reverse Transcriptase were purchased from Invitrogen (Carlsbad, CA, USA). Calcium phosphate (CaP) coated plates were purchased from Cosmo Bio (Tokyo, Japan). All other chemicals were purchased from Sigma (St. Louis, MO, USA) unless otherwise indicated [24]. RT-PCR primers are listed in Table 1.

### 4.2. Animal Ethics and Bone Marrow Macrophage Cell Culture

The study was approved by the ethics committee of the animal handling, Jeonbuk National University, South Korea (CBNU 2020-050; 6 May 2020). The tibiae and femur of 6-week male ICR mice were used to obtain BMMs. In addition, they were seeded with α-MEM, 2 mM L-glutamate, 10% FBS, 100 U/mL penicillin, and 100 μg/mL streptomycin in 5% CO_2_ at 37 °C, 30 ng/mL M-CSF for 3 days. After 3 days, 1X PBS was used to wash the BMM cells, and adherent cells were used for further experiments.

### 4.3. Cell Viability

BMMs (4 × 10^3^ cells/well) were seeded in 96-well plates and incubated overnight in culture media containing 10% FBS. Then, different concentrations of KT were used to treat cells for 72 h. Cell viability measurements were done as described previously [24].

### 4.4. Osteoclast Differentiation

Cells (2 × 10^3^ cells/well) were added to a 96-well plate, then 30 ng/mL M-CSF was mixed in a complete media and incubated on the plate. RANKL (100 ng/mL) was added with several concentrations of KT. The mixture was changed every 2 days. After 5 days, the tartrate-resistant acid phosphatase (TRAP) activity was measured as previously described [24].

### 4.5. Resorption Pit Area

BMMs (3 × 10^3^ cells/well) were added in calcium phosphate (CaP) coated 48-well plates in 30 ng/mL M-CSF, 100 ng/mL RANKL, and the presence or absence of KT at different concentrations for 6 days. After 6 days, the fluorescence intensity was measured, and the pit area was calculated as defined earlier [24].

### 4.6. Fibrous Actin Ring Formation

After differentiation, BMM cells were washed with 1 × PBS three times, 4% formaldehyde was added for 15 min at room temperature, washed with PBS, and permeabilized in 0.1% Titron for 1 min. 1 × Red Fluorescent Phalloidin Conjugate was added for 60 min, after which the cells were washed with 1 × PBS three times, and 4′,6-diamidino-2-phenylindole (DAPI) was added for nuclear staining. Finally, a fluorescence microscope (Olympus) was used to visualize actin ring formation.

### 4.7. RNA Extraction and Reverse-Transcribed mRNA

Total RNA was acquired from BMM cells using TRIZOL reagents. The superscript synthesis system (Invitrogen) was used to synthesize cDNA using 1 µg of RNA. Specific primer sequences are listed below in Table 1. Reverse transcribed mRNA expression was achieved as described earlier [23].

### 4.8. Immunoblot

BMM cells were lysed in a lysis buffer, 10% SDS-PAGE was used to separate the protein, and those proteins were transferred to a polyvinylidene difluoride membrane using a transfer buffer. Nonfat skim milk (5%) was prepared, and the membranes were blocked for 60 min at room temperature. The blocked membranes were treated with primary rabbit anti-p-ERK, anti-p-p38, anti-p-JNK, anti-ERK, anti-JNK, anti-p38, anti-NFATc1, anti-cathepsin K, and anti-β-actin antibodies, which were diluted at 1:500 to 1:1000 ratios and incubated at 4 °C for 15 h. Horseradish peroxidase-conjugated anti-rabbit or anti-mouse secondary antibodies were diluted at a 1:3000 to 1:5000 ratio in 5% skim milk for 2 h at room temperature. Protein expression was then determined with an ECL detection kit (Bio-Rad, Hercules, CA, USA) and exposure to an X-ray film.

### 4.9. In Vivo Experiments

ICR mice (6 weeks age) were divided into four groups of 10 mice each. PBS was used for the negative control group and LPS formed the positive control group (5 mg/Kg). LPS + a low dose of KT (1 mg/kg), and LPS + high dose KT (5 mg/kg) formed the two treatment groups. PBS or KT was provided orally to mice every 48 h for 10 days. LPS (5 mg/kg) was injected intraperitoneally 1 day after the drug treatment and three times injected before sacrifice. The mice were sacrificed after 10 days, and bone loss was detected using microcomputed tomography (μCT) and histopathology.

### 4.10. U-CT and Histopathology

The left femur of each treated mouse was scanned with a SkyScan 1076 μCT scanner. The settings for the X-way tube were 100 kV voltage and 100 μA current with a 240 ms exposure time to obtain an image. For histological analysis, 10% formalin was used to fix the right femurs of each mouse at room temperature overnight. EDTA (12%, pH 7.4) was used to decalcify the femurs for 1 month, at which time they were embedded in paraffin. Paraffin slices (5 µm) were sectioned and stained with hematoxylin-eosin (H&E) staining and TRAP staining as previously described [24].

### 4.11. Statistical Analysis

Experiments were performed three times and analyzed by one-way ANOVA with Holm-Sidak’s multiple comparisons test. The Graphpad Prism software was used to perform all statistical tests (Graphpad Software Inc., La Jolla, CA, USA), and the data are expressed as mean ± SD. Values of *P* < 0.05 were considered statistically significant.

## Figures and Tables

**Table 1:** Primer sequences and conditions for RT-PCR.

Target Genes (Accession Number)	Primer (Forward, Reverse)	Annealing Tm (°C)	PCR Cycles
TRAP (NM_007388)	5′-ctgctgggcctacaaatcat-3′ 5′-ggtagtaagggctggggaag-3′	54	30
MMP9 (NM_013599)	5′-cgtcgtgatccccacttact-3′ 5′-agagtactgcttgcccagga-3′	57.5	36
Cathepsin K NM_007802)	5′-aggcggctatatgaccactg-3′ 5′-ccgagccaagagagcatatc-3′	57.5	26
c-Fos (NM_010234)	5′-atgggctctcctgtcaacac-3′ 5′-ggctgccaaaataaactcca-3′	57.5	30
NFATc1 (NM_198429) DC-STAMP (AY517483)	5′-gggtcagtgtgaccgaagat-3′ 5′-aggtgggtgaagactgaagg-3′ 5′-ctaaggagaagaaacccttg-3′ 5′-cagcatagaagacaacaatcc-3′	55 54	35 35
β-actin (NM_007393)	5′-ttctacaatgagctgcgtgt-3′ 5′-ctcatagctcttctccaggg-3′	50	26

## Data Availability

The data presented in this study are available on request from the corresponding author. The data are not publicly available due to privacy.

## Supplementary Materials

The following are available online at https://www.mdpi.com/1422-0067/22/5/2303/s1, Table S1: Parameters of bone from µCT and histomorphometry.
