# Supplementary material for: Kalkitoxin Reduces Osteoclast Formation and Resorption and Protects against Inflammatory Bone Loss
Source: Int J Mol Sci. 2021 Feb 25;22(5):2303. doi: 10.3390/ijms22052303 (PMC7956546; doi:10.3390/ijms22052303)
Supplement: Supplementary file 1 [file ijms-22-02303-s001.pdf]

Table S1 Parameters of bone from µCT and histomorphometry

Parameters	Sham	LPS	LPS + L. KT	LPS + H. KT
Trabecular bone, µCT[a]				
Trabecular BMD (g/cm^3)	0.31 ±0.07	0.22 ± 0.04 #	0.23 ± 0.03 *	0.29 ± 0.03 **
Bone volume/total volume, %	35.31 ± 3.27	19.47 ± 3.52 #	28.65 ± 3.08 *	33.45 ± 2.51 **
Trabecular number[b]	2.81 ± 0.16	1.93 ± 0.23 #	2.25 ± 0.34	2.70 ± 0.17 *
Trabecular thickness, mm	0.55 ± 0.64	0.32 ± 0.51 #	0.41 ± 0.26 *	0.49 ± 0.42 **
Trabecular separation, mm	0.35 ± 0.06	0.51 ± 0.04 #	0.41 ± 0.03 *	0.33 ± 0.05 **
Cortical bone, µCT				
Cortical BMD	1.55 ± 0.65	1.22 ± 0.44 #	1.25 ± 0.31 *	1.35 ± 0.39 **
Cortical total area, mm^2	10.55 ± 0.84	8.91 ± 0.25 #	9.68 ± 0.77 *	10.08 ± 0.19 **
Cortical bone area, mm^2	5.51 ± 0.31	4.632 ± 0.76 #	5.31 ± 0.64 *	5.37 ± 0.34 *
Cortical marrow area, mm^2	5.04 ± 0.30	4.27 ± 0.63 #	4.37 ± 0.30 *	4.70 ± 0.26 **
Cortical thickness, mm	0.40 ± 0.42	0.34 ± 0.33 #	0.36 ± 0.16	0.39 ± 0.27 *
Trabecular bone, histomorphometry[c]				
Osteoclast surface/bone surface, %	7.5 ± 0.75	29 .3 ± 4.2 #	20.4 ± 2.3 *	12.6 ± 1.9 **
Osteoclast number[d]	5.3 ± 0.92	26.2 ± 3 #	18.7 ± 1.5 *	9.4 ± 1.2 **

Note: Values are the mean ± SD of at least 10 animals per group. LPS = Lipopolysaccharide

Mean ± SD, $^{*}P < 0.05$ vs. LPS treated (#)

[a]Proximal femoral metaphysis.

[b]Number of plates per unit of length 9mm.

[c]Proximal femoral metaphysis, 100 µm from distal end of growth plaste excluding the endocortical surfaces, longitudinal sections.

[d]Number of osteoclasts per mm^2 of bone surface.
